# Dietary supplementation with olive mill wastewaters induces modifications on chicken jejunum epithelial cell transcriptome and modulates jejunum morphology

**DOI:** 10.1186/s12864-018-4962-9

**Published:** 2018-08-02

**Authors:** Marcella Sabino, Katia Cappelli, Stefano Capomaccio, Luisa Pascucci, Ilaria Biasato, Andrea Verini-Supplizi, Andrea Valiani, Massimo Trabalza-Marinucci

**Affiliations:** 1 Department of Veterinary Medicine, University of Perugia, Via San Costanzo 4, 06126 Perugia, Italy; 2 Department of Veterinary Sciences, University of Torino, Largo Paolo Braccini 2, 10095 Grugliasco, Italy; 3 Istituto Zooprofilattico Sperimentale dell’Umbria e delle Marche, Via Gaetano Salvemini 1, 06126 Perugia, Italy

**Keywords:** Nutrigenomics, Differentially expressed genes, Anti-viral activity, Cholesterol biosynthesis, Fatty acid metabolism

## Abstract

**Background:**

The Mediterranean diet is considered one of the healthier food habits and olive oil is one of its key components. Olive oil polyphenols are known to induce beneficial effects in several pathological conditions, such as inflammatory bowel disease, and to contrast the proliferation of cancer cells or hypercholesterolemia. Polyphenols are also present in waste products derived from the olive industry: olive mill wastewaters (OMWW) are rich in polyphenols and there is an increasing interest in using OMWW in animal nutrition. OMWW are attributed with positive effects in promoting chicken performance and the quality of food-derived products. However, a tissue-specific transcriptome target analysis of chickens fed with OMWW has never been attempted.

**Results:**

We explored the effect of dietary OMWW on the intestinal function in broilers. A morphological analysis of the jejunum revealed that OMWW reduced crypt depth, whereas no significant modifications were observed for villus height and the villus height/crypt depth ratio. An RNA Sequencing analysis was performed on isolated, intestinal, epithelial cells and 280 differentially expressed genes were found using a count-based approach. An enrichment analysis revealed that the majority of up regulated genes in the OMWW group were over-represented by the regulation of viral genome replication-related GO-Terms, whereas down regulated genes were mainly involved in cholesterol and lipid metabolism.

**Conclusions:**

Our study showed how an industrial waste product can be recycled as a feed additive with a positive relapse. OMWW dietary supplementation can be a nutritional strategy to improve chicken performance and health, prevent intestinal damage, enhance innate immunity and regulate cholesterol metabolism and fat deposition.

**Electronic supplementary material:**

The online version of this article (10.1186/s12864-018-4962-9) contains supplementary material, which is available to authorized users.

## Background

The Mediterranean diet is the foundation of the cultural identity of the Mediterranean region and is widely recognised for its potential effects in reducing the risk of cancer and cardiovascular, metabolic and neurodegenerative diseases [1, 2]. Many beneficial properties of the Mediterranean diet appear to be related to the high consumption of olive oil. Olive oil is enriched with hydrophilic phenolic compounds, including phenolic acids and derivatives (e.g gallic acid and vanillic acid), flavones (e.g. luteolin), lignans (e.g pinoresinol), secoiridoids (e.g. oleuropeinaglycon), phenolic alcohols (e.g. hydroxytyrosol, tyrosol) [3, 4]. Indeed, the increasing interest in olive oil polyphenols is associated with their biological activities: antioxidant, antiatherogenic, antihepatotoxic, hypoglycemic, anti-inflammatory, antitumor, antiviral and immunomodulating [5–7]. For instance, oleuropein, hydroxytyrosol, tyrosol and caffeic acid are considerable scavengers of reactive oxygen and nitrogen species (ROS and RNS) [8]. Hydroxytyrosol also has potential anti-inflammatory properties, reducing pro-inflammatory signalling in human monocytes [9]. Olive oil polyphenols show versatile properties in metabolic diseases: it has been reported that oleuropein and hydroxytyrosol combat obesity, by reducing the intracellular deposit of triglyceride and decreasing the expression of genes related to the adipogenesis pathway [10–12]. Moreover, olive polyphenols have been reported to reduce glycaemia and cholesterolemia [13]. Olive oil polyphenols have a beneficial effect on the cancer cell line model: in vitro studies reveal that pinoresinol inhibits the proliferation of colon and prostate tumor cells and induces apoptosis in human leukaemia cells [14] Oleuropein, in addition, is capable of preventing colon rectal cancer in mice [15].

Worthy of note is the report that polyphenols help fight inflammatory bowel diseases. In vivo studies state that olive oil phenols prevent colitis in mice [16–18], particularly by activating PPAR signalling, the down regulation of NF- κB signalling and iNOS expression [17, 18]. In intestinal epithelial cells (Caco-2) exposed to inflammatory stimuli, treatment with polyphenols reduces the expression of *IL-8* and *NF-κB* and also affects *IL8* mRNA stability by regulating post-transcriptional signalling [19]. These studies clearly state that these compounds can act directly on intestinal epithelial cells, which play an active role against invading pathogens in an immune response and in gastrointestinal tract functions [19–21].

Olive oil polyphenol compounds have also been found in olive mill wastewaters (OMWW), one of the waste products obtained during the olive oil extraction process [22], with a high percentage of hydroxytyrosol, tyrosol, verbascoside and other aglycon derivatives [23–26]. OMWW polyphenols are correlated to antiviral, antibacterial and antifungal activities and they are known to play a role in preventing cardiovascular diseases and tumor progression [27–30]. OMWW polyphenols also possess antioxidant effects on human, intestinal, epithelial cells [31] and show hypoglycaemic effects in diabetic rats [32]. Interestingly, the use of OMWW dietary supplementation is increasing and the aim is to promote animal performance and the quality of derived products. For instance, OMWW dietary supplementation improves the redox status of broilers, by reducing both protein and lipid oxidation and enhancing the activity of antioxidant enzymes [33]. Moreover, the OMWW extract has been proved effective against *P. fluorescens,* which is responsible for the negative, organoleptic properties of mozzarella cheese [26], and has, therefore, been proposed as a functional ingredient in milk for its role in reducing Maillard reaction products [34]. OMWW polyphenols have been also effective in reducing faecal shedding of *Campylobacter* spp. in broilers [35], which is of particular interest considering that a high number of foodborne disease outbreaks in humans are due to the presence of *Campylobacter* spp. in poultry meat [36].

Another discovery worthy of support is that olive oil polyphenols have been found in a blend of OMWW and olive cake known as “paté”, another olive oil extraction waste product. Paté has also been used as a supplement for poultry and reports have shown its beneficial effects in improving chicken performance and the oxidative status of meat [37].

However, very little is known about the effects of OMWW on the jejunum in broilers and no modern approaches using Next Generation Sequencing techniques have been applied to such a system.

Thus, we propose a nutrigenomic investigation of the effects of a dietary supplement of OMWW on the broiler’s jejunum. A morphological characterisation and whole transcriptome analysis of intestinal epithelial cells was applied in order to detect possible changes induced by OMWW dietary supplementation.

## Results

### Morphological analysis of jejunum epithelial cells

**Fig. 1:**
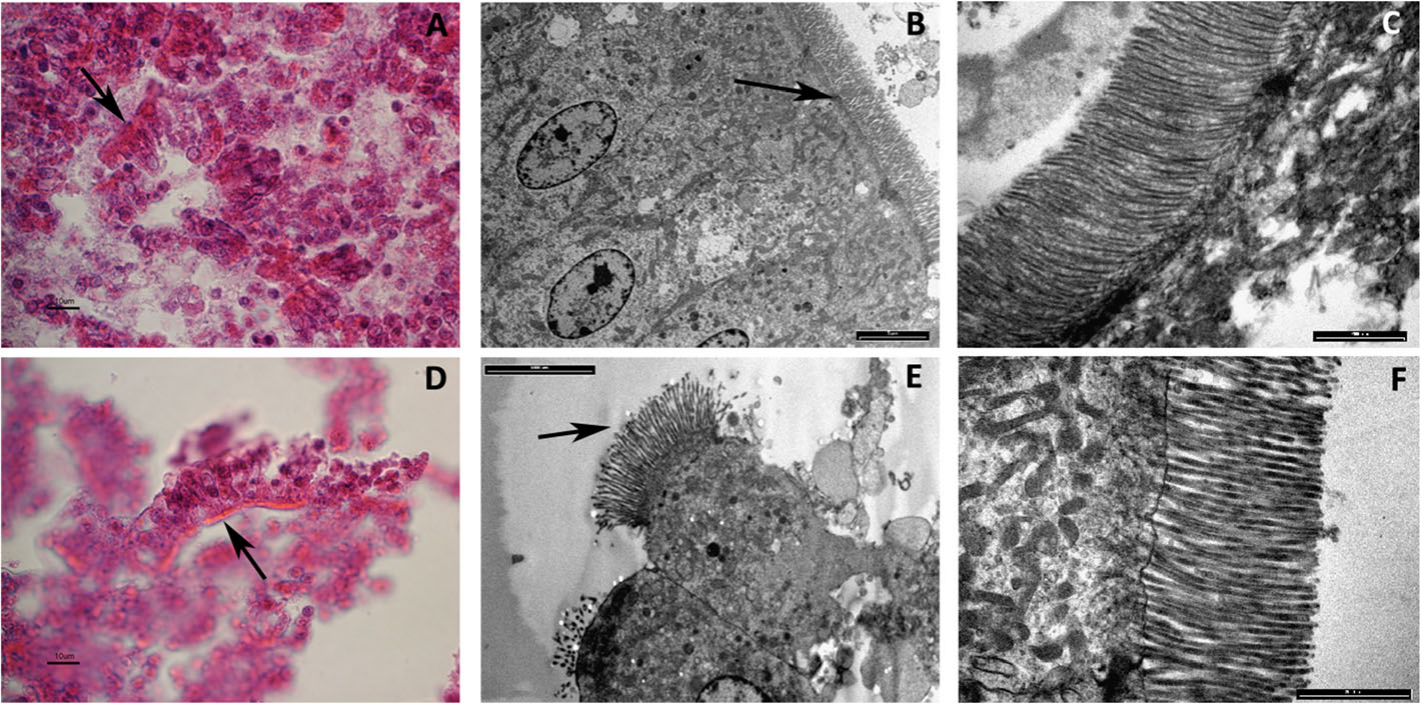
Light and transmission electron microscopy features of jejunum epithelial cells obtained by collagenase digestion. **a** and **d**. Single and grouped epithelial cells of control (**a**) and treated animals (**d**). Note the columnar shape and the typical eosinophilic brush border (arrow). Haematoxylin-Eosin, scale bar: 10 μm. **b** and **e**. Epithelial cell strips obtained by digestion of control (**b**) and treated jejunum tracts (**e**). The medium power view reveals the single layer of polarized columnar cells that cover the villous surface. The tall nuclei are lined up at the base of the cells while the apical surface is covered by microvilli (arrow). Transmission electron microscopy. Scale bar, 5000 nm. **c** and **f**. High power view of the luminal portion of the epithelial cells obtained by digestion of control (**c**) and treated jejunum tracts (**f**). Observe the surface of the columnar epithelial cells with the “brush border” consisting of closely packed microvilli. Transmission electron microscopy. Scale bar, 5000 nm

Light and transmission electron microscopy analyses confirmed the epithelial nature of recovered cells. As shown in Fig. 1, collagenase digestion resulted in the isolation of strips of tall cells, consisting of the simple columnar epithelium covering the intestinal villous surface. Columnar cells typically displayed the brush border, formed by closely packed microvilli.

The ultra-structural evaluation of cells collected from control and treated animals did not reveal any difference in terms of subcellular features. The brush border was similarly organised and developed in both groups (Fig. 1).

### Histomorphological investigations

The intestinal morphology of broiler chickens is summarised in Table 1. OMWW inclusion in the diet did not significantly affect (*P* > 0.05) the villus height (Vh) and villus height to crypt depth (Vh/Cd) ratio. On the contrary, broilers fed with OMWW showed a lower Cd (*P* < 0.01) compared to control group (CTRL).

**Table 1:** Effects of OMWW inclusion on intestinal morphometric indexes of broiler chickens (*n* = 10/treatment)

	CTRL	OMWW	SEM	*P*-value
Vh _(mm)_	1.01	0.90	0.05	0.234
Cd _(mm)_	0.07^a^	0.06^b^	0.00	0.004
Vh/Cd _(mm/mm)_	14.56	15.87	0.74	0.392

Different superscript letters (a, b) in the same row mean significant differences (*P* < 0.05) among the dietary treatments. Control, CTRL; Olive oil mill wastewaters, (OMWW); Vh, villus height; Cd, crypt depth; Vh/Cd ratio, villus height to crypt depth ratio

### RNA sequencing data analysis

The RNA sequencing experiment produced an average of 23 million read pairs per sample. Raw data are published in SRA with accession numbers from SAMN08940088 to SAMN08940106. An average of 18.3 million reads was obtained after trimming and an average of 15.1 million reads (82%) was uniquely mapped to the chicken reference genome (*Gallus gallus* v.*5*.0), with a good representation of the medium-highly expressed genes in the target tissue. Only these reads were used for the differential gene expression assessment to avoid introducing expression bias via a multi-mapper assignment. Detailed statistics on quality control and mapping are shown in Table 2.

**Table 2:** RNA Sequencing libraries details

Sample name	Raw reads	Trimmed	Uniquely Mapped	Uniquely Mapped (%)
CTRL 1	37.039.265	30.103.595	25.238.297	83.84%
CTRL 2	28.864.090	23.311.634	19.394.421	83.20%
CTRL 3	26.175.781	21.414.954	18.099.690	84.52%
CTRL 4	23.819.919	17.978.157	14.694.053	81.73%
CTRL 5	27.269.370	21.304.535	17.945.240	84.23%
CTRL 6	24.754.841	20.179.523	17.070.409	84.59%
CTRL 7	24.595.313	19.973.641	17.020.911	85.22%
CTRL 8	23.335.827	18.507.845	14.691.888	79.38%
CTRL 9	17.593.049	13.053.838	10.563.297	80.92%
CTRL 10	15.202.953	10.908.028	8.974.349	82.27%
OMWW 1	16.047.903	11.408.761	9.497.639	83.25%
OMWW 2	18.438.802	13.501.457	11.200.681	82.96%
OMWW 3	18.519.366	15.975.728	12.945.322	81.03%
OMWW 5	17.782.725	15.398.684	11.865.229	77.05%
OMWW 6	18.273.935	15.835.585	12.952.124	81.79%
OMWW 7	26.686.925	22.289.512	19.036.201	85.40%
OMWW 8	21.648.754	18.174.251	15.709.291	86.44%
OMWW 9	27.390.420	21.368.047	17.413.426	81.49%
OMWW 10	28.732.976	21.833.209	17.909.295	82.03%

### Differentially expressed genes

After a statistical analysis with edgeR using a data set of 9162 filtered genes, we found 280 differentially expressed genes (DEG) in the isolated, epithelial cells of the jejunum in the OMWW group compared to the CTRL group, with a significance of adjusted *p*-value (q-value) < 0.05 and an absolute log fold change (logFC) of over 1.0. Using these filters, 139 genes were up regulated (logFC > 1.0), whereas 141 genes were down- regulated (logFC > − 1.0). After annotating the differential expressed genes using BioMart, the associated gene names were used to perform an enrichment analysis. All details are shown in the Additional file 1.

### Gene functional analysis

The annotated list from the differential gene expression analysis was used as input for the gene ontology enrichment and pathway analysis. The enrichment according to the three biological vocabularies (Cellular Component, Biological Process and Molecular function) and Kyoto Encyclopaedia of Genes and Genomes (KEGG) pathways was assessed using ClueGO, a Cytoscape plugin.

Most of the down regulated DEGs were over-represented in KEGG pathways (*PPAR signalling pathway, Steroid biosynthesis)* or GO terms related to lipid metabolism (*fatty acid metabolic process, triglyceride metabolic process, cholesterol biosynthetic process, lipid biosynthetic process, phospholipid biosynthetic process, steroid biosynthetic process, and sterol metabolic process*).

We also found a massive up regulation of genes enriched for the GO-Terms (*regulation of viral process, regulation of viral life cycle, viral genome replication, regulation of viral genome replication, negative regulation of viral process, response to virus, negative regulation of viral life cycle, negative regulation of viral genome replication*) and the KEGG pathway (*Influenza A)* of the viral process. All details are reported in Tables 3 and 4.

**Table 3:** Significantly enriched GO Terms for the three vocabularies obtained via ClueGO (FDR < 0.05)

GO-ID	GO-Term	FDR	Nr. Genes	Associated Genes Found
GO:0006066	alcohol metabolic process	2,30E-04	11,00	[ABHD3, CHDH, CHPT1, CYP51A1, DHCR24, ENPP7, FDPS, HMGCS1, INSIG1, MSMO1, NSDHL]
GO:0016126	sterol biosynthetic process	2,70E-04	5,00	[CYP51A1, FDPS, HMGCS1, INSIG1, MSMO1]
GO:0000793	condensed chromosome	3,30E-04	8,00	[BRCA1, CENPW, MSH4, NDC80, NEK2, PLK1, SGOL1, SMC2]
GO:1901615	organic hydroxy compound metabolic process	3,40E-04	13,00	[ABHD3, ALDH9A1, BBOX1, CHDH, CHPT1, CYP51A1, DHCR24, ENPP7, FDPS, HMGCS1, INSIG1, MSMO1, NSDHL]
GO:0008610	lipid biosynthetic process	3,70E-04	14,00	[AGMO, BRCA1, CHPT1, CYP51A1, FDFT1, FDPS, GPAM, HMGCS1, INSIG1, MSMO1, NSDHL, PIGA, PLD1, TCF7L2]
GO:0016125	sterol metabolic process	3,80E-04	7,00	[CYP51A1, DHCR24, FDPS, HMGCS1, INSIG1, MSMO1, NSDHL]
GO:0000794	condensed nuclear chromosome	4,00E-04	6,00	[BRCA1, MSH4, NDC80, NEK2, PLK1, SGOL1]
GO:0008203	cholesterol metabolic process	6,20E-04	6,00	[CYP51A1, DHCR24, FDPS, HMGCS1, INSIG1, NSDHL]
GO:1902652	secondary alcohol metabolic process	6,90E-04	6,00	[CYP51A1, DHCR24, FDPS, HMGCS1, INSIG1, NSDHL]
GO:0006695	cholesterol biosynthetic process	7,20E-04	4,00	[CYP51A1, FDPS, HMGCS1, INSIG1]
GO:1902653	secondary alcohol biosynthetic process	7,20E-04	4,00	[CYP51A1, FDPS, HMGCS1, INSIG1]
GO:0006577	amino-acid betaine metabolic process	1,10E-03	3,00	[ALDH9A1, BBOX1, CHDH]
GO:0006631	fatty acid metabolic process	1,10E-03	9,00	[AACS, AGMO, BRCA1, ETFA, GPAM, HADHA, INSIG1, MSMO1, SLC27A4]
GO:0051297	centrosome organization	1,20E-03	6,00	[BRCA1, HAUS8, NEK2, NPM1, PLK1, SGOL1]
GO:0032787	monocarboxylic acid metabolic process	1,20E-03	12,00	[AACS, AGMO, ALDH9A1, BBOX1, BRCA1, ETFA, GPAM, HADHA, INSIG1, MSMO1, SLC27A4, VNN1]
GO:0098813	nuclear chromosome segregation	1,30E-03	8,00	[MSH4, NDC80, NEK2, NUSAP1, PLK1, SGOL1, SMC2, UBE2C]
GO:0007059	chromosome segregation	1,50E-03	9,00	[BRCA1, MSH4, NDC80, NEK2, NUSAP1, PLK1, SGOL1, SMC2, UBE2C]
GO:0000780	condensed nuclear chromosome, centromeric region	1,50E-03	3,00	[NDC80, PLK1, SGOL1]
GO:0031023	microtubule organizing center organization	1,50E-03	6,00	[BRCA1, HAUS8, NEK2, NPM1, PLK1, SGOL1]
GO:0046165	alcohol biosynthetic process	1,60E-03	6,00	[CHPT1, CYP51A1, FDPS, HMGCS1, INSIG1, MSMO1]
GO:0006576	cellular biogenic amine metabolic process	1,60E-03	5,00	[ABHD3, CHDH, CHPT1, ENPP7, SMOX]
GO:0045132	meiotic chromosome segregation	1,90E-03	4,00	[MSH4, PLK1, SGOL1, SMC2]
GO:0042439	ethanolamine-containing compound metabolic process	2,20E-03	4,00	[ABHD3, CHDH, CHPT1, ENPP7]
GO:1901617	organic hydroxy compound biosynthetic process	2,20E-03	7,00	[BBOX1, CHPT1, CYP51A1, FDPS, HMGCS1, INSIG1, MSMO1]
GO:0000070	mitotic sister chromatid segregation	2,30E-03	6,00	[NDC80, NEK2, NUSAP1, PLK1, SMC2, UBE2C]
GO:0097164	ammonium ion metabolic process	2,70E-03	6,00	[ABHD3, ALDH9A1, BBOX1, CHDH, CHPT1, ENPP7]
GO:0008608	attachment of spindle microtubules to kinetochore	2,80E-03	3,00	[NDC80, NEK2, SGOL1]
GO:0000779	condensed chromosome, centromeric region	3,10E-03	4,00	[CENPW, NDC80, PLK1, SGOL1]
GO:0045071	negative regulation of viral genome replication	3,10E-03	3,00	[EIF2AK2, OASL, RSAD2]
GO:0009308	amine metabolic process	4,00E-03	5,00	[ABHD3, CHDH, CHPT1, ENPP7, SMOX]
GO:0003725	double-stranded RNA binding	4,00E-03	4,00	[DHX58, EIF2AK2, OASL, TLR3]
GO:0008202	steroid metabolic process	4,00E-03	7,00	[CYP51A1, DHCR24, FDPS, HMGCS1, INSIG1, MSMO1, NSDHL]
GO:0044106	cellular amine metabolic process	4,10E-03	5,00	[ABHD3, CHDH, CHPT1, ENPP7, SMOX]
GO:0000281	mitotic cytokinesis	4,10E-03	3,00	[MITD1, NUSAP1, PLK1]
GO:0009615	response to virus	4,60E-03	8,00	[DDX60, DHX58, EIF2AK2, GPAM, IKBKE, OASL, RSAD2, TLR3]
GO:0000819	sister chromatid segregation	4,70E-03	6,00	[NDC80, NEK2, NUSAP1, PLK1, SMC2, UBE2C]
GO:0000776	kinetochore	4,90E-03	5,00	[CENPW, NDC80, NEK2, PLK1, SGOL1]
GO:1904030	negative regulation of cyclin-dependent protein kinase activity	7,70E-03	3,00	[NPM1, PLK1, UBE2C]
GO:0008654	phospholipid biosynthetic process	8,40E-03	5,00	[CHPT1, FDPS, GPAM, PIGA, PLD1]
GO:0061640	cytoskeleton-dependent cytokinesis	8,50E-03	3,00	[MITD1, NUSAP1, PLK1]
GO:0000777	condensed chromosome kinetochore	9,10E-03	3,00	[CENPW, NDC80, PLK1]
GO:0045840	positive regulation of mitotic nuclear division	9,10E-03	3,00	[NUSAP1, PLK1, UBE2C]
GO:0072330	monocarboxylic acid biosynthetic process	1,00E-02	5,00	[AGMO, BBOX1, BRCA1, INSIG1, MSMO1]
GO:0046486	glycerolipid metabolic process	1,20E-02	7,00	[ABHD3, CHPT1, GPAM, INSIG1, PIGA, PLD1, TCF7L2]
GO:0044242	cellular lipid catabolic process	1,30E-02	5,00	[ENPP7, ETFA, GALC, HADHA, SLC27A4]
GO:0098661	inorganic anion transmembrane transport	1,30E-02	4,00	[ANO6, LOC101748788, SLC20A1, SLC26A2]
GO:1903901	negative regulation of viral life cycle	1,40E-02	3,00	[EIF2AK2, OASL, RSAD2]
GO:0008081	phosphoric diester hydrolase activity	1,40E-02	4,00	[ENPP7, GDPD1, PDE9A, PLD1]
GO:0051785	positive regulation of nuclear division	1,40E-02	3,00	[NUSAP1, PLK1, UBE2C]
GO:0051607	defense response to virus	1,50E-02	6,00	[DDX60, DHX58, GPAM, OASL, RSAD2, TLR3]
GO:0006633	fatty acid biosynthetic process	1,50E-02	4,00	[AGMO, BRCA1, INSIG1, MSMO1]
GO:0015103	inorganic anion transmembrane transporter activity	1,50E-02	4,00	[ANO6, LOC101748788, SLC20A1, SLC26A2]
GO:0045069	regulation of viral genome replication	1,50E-02	3,00	[EIF2AK2, OASL, RSAD2]
GO:0000922	spindle pole	1,50E-02	4,00	[NEK2, NPM1, PLK1, SGOL1]
GO:0048806	genitalia development	1,60E-02	3,00	[DHCR24, KLHL10, TCF7L2]
GO:0048525	negative regulation of viral process	1,70E-02	3,00	[EIF2AK2, OASL, RSAD2]
GO:1902850	microtubule cytoskeleton organization involved in mitosis	1,80E-02	3,00	[NDC80, NEK2, PLK1]
GO:0000775	chromosome, centromeric region	1,90E-02	5,00	[CENPW, NDC80, NEK2, PLK1, SGOL1]
GO:0022626	cytosolic ribosome	2,10E-02	4,00	[RP11-849F2.7, RPL21, RPL9, RPS23]
GO:0043901	negative regulation of multi-organism process	2,10E-02	4,00	[DHX58, EIF2AK2, OASL, RSAD2]
GO:1901989	positive regulation of cell cycle phase transition	2,10E-02	3,00	[NPM1, PLK1, UBE2C]
GO:0006641	triglyceride metabolic process	2,10E-02	3,00	[GPAM, INSIG1, TCF7L2]
GO:0090068	positive regulation of cell cycle process	2,20E-02	5,00	[BRCA1, NPM1, NUSAP1, PLK1, UBE2C]
GO:0009062	fatty acid catabolic process	2,30E-02	3,00	[ETFA, HADHA, SLC27A4]
GO:0016614	oxidoreductase activity, acting on CH-OH group of donors	2,30E-02	4,00	[CHDH, DHCR24, HADHA, NSDHL]
GO:0035725	sodium ion transmembrane transport	2,30E-02	4,00	[ANO6, CNKSR3, SLC20A1, STOML1]
GO:0019079	viral genome replication	2,30E-02	3,00	[EIF2AK2, OASL, RSAD2]
GO:0007126	meiotic nuclear division	2,40E-02	4,00	[MSH4, PLK1, SGOL1, SMC2]
GO:0022625	cytosolic large ribosomal subunit	2,60E-02	3,00	[RP11-849F2.7, RPL21, RPL9]
GO:0045017	glycerolipid biosynthetic process	2,60E-02	4,00	[CHPT1, PIGA, PLD1, TCF7L2]
GO:0006639	acylglycerol metabolic process	2,70E-02	3,00	[GPAM, INSIG1, TCF7L2]
GO:0045444	fat cell differentiation	2,80E-02	5,00	[FNDC5, INSIG1, PEX11A, SOCS1, TCF7L2]
GO:0015698	inorganic anion transport	2,80E-02	4,00	[ANO6, LOC101748788, SLC20A1, SLC26A2]
GO:0006638	neutral lipid metabolic process	2,80E-02	3,00	[GPAM, INSIG1, TCF7L2]
GO:0007051	spindle organization	2,80E-02	4,00	[HAUS8, NDC80, NEK2, PLK1]
GO:0007098	centrosome cycle	3,00E-02	3,00	[BRCA1, NEK2, NPM1]
GO:0005254	chloride channel activity	3,00E-02	3,00	[ANO6, LOC101748788, SLC26A2]
GO:1903046	meiotic cell cycle process	3,00E-02	4,00	[MSH4, PLK1, SGOL1, SMC2]
GO:0007052	mitotic spindle organization	3,30E-02	3,00	[NDC80, NEK2, PLK1]
GO:0051092	positive regulation of NF-kappaB transcription factor activity	3,30E-02	3,00	[EIF2AK2, NPM1, TLR3]
GO:0004386	helicase activity	3,40E-02	4,00	[DDX60, HELB, MOV10, PIF1]
GO:0072329	monocarboxylic acid catabolic process	3,40E-02	3,00	[ETFA, HADHA, SLC27A4]
GO:0004620	phospholipase activity	3,40E-02	3,00	[ABHD3, ENPP7, PLD1]
GO:0051983	regulation of chromosome segregation	3,40E-02	3,00	[NEK2, PLK1, UBE2C]
GO:0010565	regulation of cellular ketone metabolic process	3,50E-02	3,00	[BRCA1, INSIG1, TCF7L2]
GO:1902476	chloride transmembrane transport	3,60E-02	3,00	[ANO6, LOC101748788, SLC26A2]
GO:0051321	meiotic cell cycle	3,60E-02	4,00	[MSH4, PLK1, SGOL1, SMC2]
GO:0051225	spindle assembly	3,60E-02	3,00	[HAUS8, NEK2, PLK1]
GO:0005253	anion channel activity	3,70E-02	3,00	[ANO6, LOC101748788, SLC26A2]
GO:2001251	negative regulation of chromosome organization	3,70E-02	3,00	[BRCA1, PIF1, PLK1]
GO:0015297	antiporter activity	3,90E-02	3,00	[LOC101748788, SLC26A2, SLC7A4]
GO:0015108	chloride transmembrane transporter activity	3,90E-02	3,00	[ANO6, LOC101748788, SLC26A2]
GO:0050660	flavin adenine dinucleotide binding	3,90E-02	3,00	[CHDH, DHCR24, ETFA]
GO:0051053	negative regulation of DNA metabolic process	4,00E-02	3,00	[ENPP7, PIF1, POLQ]
GO:1904029	regulation of cyclin-dependent protein kinase activity	4,50E-02	3,00	[NPM1, PLK1, UBE2C]
GO:0008286	insulin receptor signaling pathway	4,80E-02	3,00	[IRS4, KL, SOCS1]

*GO-ID* GO term accession number, *GOTerm* name of Gene Ontology Term, *FDR* (False Discovery Rate) after Benjamini-Hochberg correction, *Nr. Genes* number of input genes found per term, *Associated Genes Found* associated name of genes found per term

**Table 4:** Significantly enriched KEGG pathways obtained via ClueGO (FDR < 0.05)

ID	KEGG pathway	FDR	Nr. Genes	Associated Genes Found
KEGG:0000100	Steroid biosynthesis	1,60E-04	5,00	[CYP51A1, DHCR24, FDFT1, MSMO1, NSDHL]
KEGG:0005164	Influenza A	4,60E-03	7,00	[EIF2AK2, FDPS, IKBKE, KPNA2, MX1, RSAD2, TLR3]
KEGG:0000280	Valine, leucine and isoleucine degradation	6,90E-03	4,00	[AACS, ALDH9A1, HADHA, HMGCS1]
KEGG:0000650	Butanoate metabolism	8,50E-03	3,00	[AACS, HADHA, HMGCS1]
KEGG:0000410	beta-Alanine metabolism	9,90E-03	3,00	[ALDH9A1, HADHA, SMOX]
KEGG:0000310	Lysine degradation	3,40E-02	3,00	[ALDH9A1, BBOX1, HADHA]
KEGG:0003320	PPAR signaling pathway	4,50E-02	3,00	[FP325317.1, MMP1, SLC27A4]

*ID* GO term accession number, *KEGG pathway* name of KEGG pathway, *FDR* (False Discovery Rate) after Benjamini-Hochberg correction, *Nr. Genes* number of input genes found per term, *Associated Genes Found* associated name of genes found per term

## Discussion

This study revealed that chicken dietary supplementation with OMWW induces changes at both a morphological and transcriptional level in the jejunum mucosa tract.

These results are of particular interest for the “feed and food” chain, considering that a waste product could be effective in promoting animal healthiness while “recycling”.

In detail, the morphological analysis revealed a significant decrease of crypt depth in the jejunal tract of the supplemented group (Table 1) that indicates a decreased turnover of the intestinal epithelium. On the contrary, deeper crypts would indicate faster tissue turnover in response to a damage of villi.

Cell migration from the crypt to the villus apex is a crucial step to balance villus epithelial shedding and maintain tissue homeostasis [38]. On the other hand, increased crypt depth in poultry is associated with small intestine damage due to stress stimuli (e.g. heat stress), which negatively influence functions in digestion and absorption [39].

These observations would allow to hypothesize that OMWW can have a protective effect on the jejunum mucosa. Performance and health status data, however, did not confirm this hypothesis, in that the two groups of birds had similar feed conversion efficiencies (average value: 2.41) and no differences in mortality and morbidity rate were recorded (data not shown). In both broilers and growing pigs, it has been observed that the beneficial effects on performance and immune response induced by plant-derived phenolic compounds are more likely to be shown when animals are under stressful environmental conditions [40, 41]. It must be emphasized that, in the present experiment, all animals were in excellent condition and showed no evidence of disease.

Moreover, the transcriptome analysis of isolated epithelial cells revealed that the incorporation of OMWW into the broiler diet was able to modulate the expression of genes mainly involved in the innate immune response to viral offenses. Compared to the CTRL group, we observed an up regulation of anti-viral genes in OMWW chickens. For example, *IKBKE* plays a crucial role in regulating antiviral signalling pathways mediated by NF-κB [42], whereas *TLR3* is involved in TLRs signalling for innate and adaptive immune responses [43]. *TLR3* is classified as a germline-encoded pattern-recognition receptor (PRR) and acts in recognising a double strand dsRNA virus [44]. The expression of *TLR3* is modulated in bowel diseases. For instance, TLR3 is down regulated in intestinal epithelial cells in patients affected by Crohn’s disease [45].

*EIF2AK2, OASL* and *MX* are known as interferon-stimulated genes (ISGs) with anti-viral activity: *EIF2AK2* is involved in dsRNA virus recognition and inhibits viral protein production [46]. An in vivo study reveals an increment of mortality of mice knock-out for *EIF2AK2* infected by West Nile Virus, which is an important zoonotic pathogen [47, 48] The *OASL* gene encodes anti-viral proteins, which hinder virus replication [49], whereas MX is a GTPase belonging to the dynamin family, which interferes with the activity of viral polymerases to contrast the virus replication cycle [50].

It has been reported that *EIF2AK2, OASL, MX* and melanoma differentiation-associated protein 5 (*MDA5*) expression is modulated by the infectious bursal disease virus, which causes a major disease with a negative economic impact in the poultry industry [42]. *MDA5* is also involved in the recognition of Avian influenza virus*,* another important cause of a high chicken mortality rate [51]. It shows anti-viral activity, in which it probably interacts with the ATP-dependent RNA helicase *(DHX58*) [51]. All these genes were significant DEG in our analysis, providing evidence that OMWW supplementation acted in modulating anti-viral genes and suggested that an increased expression of anti-viral genes could be effective in contrasting virus replication and act as a mechanism to elude a host innate immune response. Infectious bursal disease virus recognition is inhibited by a viral protein (VP3), which prevents *MDA5* binding with the viral genome [52]. On the other hand, the coronavirus strategy to evade host defences is to establish a concentration-dependent competition between the viral proteins and host proteins in favour of the virus, by reducing the transcription of host anti-viral genes [53]. We could speculate that an increment of the host protein expression mediated by an OMWW supplementation might revert the viral-host protein ratio in favour of the host.

Another mechanism to limit viral replication by the host is to modify the cell membrane lipids, which restrict the virus budding process. Interestingly, the *RSAD2* gene, up regulated in the OMWW group, appears to use precisely this mechanism [54] to limit West Nile Virus replication [55] and a wide range of other viruses, such as hepatitis C, HIV, the influenza virus and human cytomegalovirus [55–59].

*RSAD2* influences the fluidity of the membrane inhibiting farnesyl diphosphate synthase (*FDPS*) activity, which plays a role in cholesterol and isoprenoid biosynthesis [60]. It is interesting to note that our DEG analysis shows *RSAD2* up regulated in the OMWW group, whereas *FDPS* is down regulated. We can suppose that a down regulation of *FDPS* could initially be related not only to a greater activity of RSAD2, but also to OMWW supplementation effects on lipid metabolism, bearing in mind that FDPS is mainly involved in cholesterol and steroid metabolism [60].

*FDPS* is actually enriched for cholesterol metabolic process, lipid, steroid and sterol biosynthetic process related GO-Terms with other down regulated DEG: (*3-Hydroxy-3-methylglutaryl-coenzyme A (CoA) synthase 1* (*HMGCS1*), *farnesyl-diphosphate farnesyltransferase 1*(*FDFT1*), *NAD(P) dependent steroid dehydrogenase-like* (*NSDHL*), *Cytochrome P450 Family 51 Subfamily A Member 1 (CYP51A1).*

*HMGCS1, NSDHL* and *FDFT1* encode 3-Hydroxy-3-methylglutaryl-CoA synthase, squalene synthase and NAD(P)H sterol dehydrogenase, respectively, which are all key enzymes involved in different steps of cholesterol biosynthesis [61, 62] and some of which are modulated in obesity [63].

Taken as a whole, these results support the hypothesis that OMWW can also affect sterol synthesis pathway-related genes. This hypothesis is intriguing, as the balance of sterol absorption and de novo synthesis regulates cholesterol homeostasis in the intestine [64]. It has been reported that after the liver, the small intestine is the second tissue to contribute to de novo sterol synthesis in rodents, whereas in other species, such as rabbits and guinea pigs, it is the most important source, with the intestinal epithelium having the greatest synthetic capacity [65–68].

The down regulation of sterol biosynthesis reported in our study, however, suggests that OMWW could be a beneficial, nutritional strategy. This is confirmed by the known hypocholesterolemic effects of polyphenol-rich, olive mill wastewaters observed in rats fed with cholesterol-rich diets [69].

Moreover, *FDPS, NSDHL* and *FDFT1* are candidate genes to regulate fat deposition in chickens: excessive fat deposition in chickens is associated with negative effects on poultry production, in terms of feed efficiency [70]. It has also been reported that *FDPS, NSDHL* and *FDFT1* are up regulated in the fat line chickens and in liver and adipose tissue in fast growing chickens [71, 72].

Worthy of note is the presence of a modulating effect on lipid metabolism in jejunum epithelial cells due to OMWW, which is supported by a down regulation of *Matrix Metalloproteinase 1 (MMP-1)* and *Fatty Acid Binding Protein 3 (FABP-3).* As shown in our analysis, we found *MMP-1* enriched PPAR signalling KEGG pathways. *MMP-1* is involved in fatty acid oxidation and its expression is usually up regulated in inflammatory bowel disease [73, 74]. *FABP-3*, on the other hand, which belongs to genes of the FABP family, is involved in the transport of fatty acids [74–76].

Overall, these results support the suggestion that OMWW could have a beneficial effect in preventing intestinal damage and in reducing fatty acid transportation, with a subsequent decrease of body fat accumulation, which represents a critical issue in the chicken industry [70].

If we also take into consideration the results from the morphological analysis, our findings support the suggestion that OMWW supplementation could have positive effect on growth performance, since intestinal health is associated with improved nutrient digestibility [77].

## Conclusion

Our results revealed that OMWW dietary supplementation in poultry farming might be a good strategy to promote a small intestine response to damage, stimulate innate immunity and improve chicken health. In addition, the down regulation of genes mainly involved in cholesterol metabolism and fatty acid transport suggests that the use of OMWW might be extended to other livestock species to regulate sterol metabolism and fat deposition. The down regulation of genes involved in lipid metabolism observed in our study suggests that the analysis of the effects of dietary OMWW on liver and adipose tissue, which are important nutrigenomic target tissues, could be a further objective of this research.

Nevertheless, given the promising results already obtained from this first study, the use of OMWW as an additive in animal diets is an important aspect to consider in terms of circular economy and environmental impact. More interestingly, these findings showed that OMWW dietary supplementation is a good strategy to reuse a waste product, by exploiting the beneficial effects associated to its polyphenol content.

## Methods

### Experimental design

A total of 102 22-day-old female broilers (Ross 308) were reared in a conventional poultry house located in Umbria region, Italy. All broilers were randomly divided in two experimental group fed with two different diets for 20 days. One group was fed with a commercial feed (CTRL), while the other one was fed with a CTRL diet supplemented with 0.03% of olive mill wastewater (OMWW). To obtain the dietary supplement to be included in the poultry feed, OMWW was processed through the use of a filtration system with progressive permeability membranes [25] and finally dehydrated using a spry-drying system. Feed analyses were performed according to AOAC [78] and metabolisable energy was calculated according to Carré and Rozo [79]. The diet details are shown in Table 5.

**Table 5:** Ingredients of the control^a^ grower-finisher diet

Ingredients (kg/100 kg)	
Maize	47.51
Soybean meal (44% crude protein)	33.95
Wheat shorts	7.00
Whole roasted soybean	6.00
Soybean oil	2.40
Calcium carbonate	1.42
Dicalcium phosphate	0.64
Mineral and vitamin premix^b^	0.50
Sodium chloride	0.30
Enzymes	0.25
Chemical composition (kg/100 kg)	
Dry matter	87.81
Crude protein	21.16
Ether extracts	6.03
Ash	5.87
Neutral detergent fiber	11.63
Acid detergent fiber	4.84
Lignin	0.82
Starch	31.81
Metabolisable energy (Kcal/kg)	3120

^a^In the OMWW diet, 0.03% of olive mill waste water was substituted for 0.03% maize

^b^Integrations per Kg of feed: vitamin A 13500 U.I.; vitamin D3 U.I. 3750; ferrous carbonate mg 93.2; anhydrous calcium iodate mg 2.3; copper sulfate pentahydrate mg 59; manganese oxide 51.6 mg; manganese sulphate monohydrate mg 123.2; zinc oxide 93 mg; sodium selenite mg 0.4

The entire trial was performed according to the European Directive 2010/63/EU on animal welfare.

### Isolation of jejunum epithelial cells and jejunum histological characterisation

At slaughter, the entire small intestine was excised and a 10 cm-long segment of jejunum was cut, in order to isolate epithelial cells. The lumen was flushed with 30 ml of washing solution, composed of sterile, ice-cold PBS, supplemented with 200 U/mL penicillin, 200 μg/mL streptomycin, 12.5  μg/mL amphotericin B (Sigma, St. Louis, MO, USA) and 10 μg/mL gentamicin (Euroclone, Milan, Italy). Both ends of each jejunum tract were clumped after being filled with pre-warmed 0.1% collagenase type I (Wortighton. Lakewood, NJ, USA). After 10 min of incubation at 37 °C, each sample was unclamped and the enzymatic solution containing mucosal epithelial cells was recovered and centrifuged at 300 g for 10 min. Cells were suspended in 1 ml of PureZOL (BioRad, CA, USA) and stored at − 80 °C until RNA isolation.

### Morphological analysis of isolated cells

Isolated cells were analysed both by light and transmission electron microscopy. For this purpose, they were fixed with 10% buffered formalin, paraffin embedded, sectioned at 5 μm thickness, and stained with haematoxylin & eosin (H&E) for light microscopy observation. For the electron microscopy analysis, isolated cells were fixed with 2.5% glutaraldehyde in 0.1 M phosphate buffer, pH 7.3, for 1 h at room temperature, post-fixed in 2% osmium tetroxide, dehydrated in a graded series of ethanol up to absolute, pre-infiltrated and embedded in Epon 812. Ultrathin sections (90 nm) were mounted on 200-mesh copper grids, stained with uranyl acetate and lead citrate, and examined by a Philips EM 208.

### Histomorphological investigations of jejunum samples

Samples of jejunum were fixed in a 10% buffered formalin solution for morphometric investigations. Tissues were routinely embedded in paraffin wax blocks, sectioned at a thickness of 5 μm, mounted on glass slides and stained with H&E. The evaluated morphometric indexes included the villus height (Vh, from the tip of the villus to the crypt), the crypt depth (Cd, from the base of the villus to the submucosa) and the villus height to crypt depth (Vh/Cd) ratio [80]. Morphometric analyses were performed on 10, well-oriented, intact villi and 10 crypts, chosen from the intestinal segments collected [81].

The statistical analysis was performed using the GraphPad® Prism software (v. 6). The Shapiro-wilk test established normal data distribution. A student’s t test was used to compare the morphometric indexes between the dietary treatments. Significance was declared at *P* < 0.05. The results were expressed as mean and pooled standard error of the mean (SEM).

### RNA extraction

The total RNA from all 20 jejunum epithelial cell samples was isolated according to the Aurum Total RNA Fatty and Fibrous Tissue kit instructions (BioRad, CA, USA). The genomic DNA from each sample was removed using DNAse treatment, according to the TURBO DNAse manufacturer’s specifications (Ambion – Life Technologies, CA, USA). In order to deactivate the DNAse activity, each sample of RNA was then purified using the phenol-chloroform-isoamyl alcohol method, according to the Sambrook et al. protocol [82].

The RNA quantity and quality were evaluated using a NanoDrop 2000 spectrophotometer (Thermo Fisher Scientific, Waltham, MA, USA) and Qubit 2.0 Flurometer (Life Technologies, MA, USA), whereas RNA integrity was carried out by microfluidic electrophoresis on a BioAnalyzer 2100 (Agilent Technologies).

The RNA Integrity Number (RIN) score of the CTRL group was ranging from 3.9 to 6.9 (mean 5.7 ± 1.1); while the OMWW RIN value was ranging from 2.3 to 5.6 (mean 4.3 ± 0.8). One sample (OMWW4) was excluded at this step because of a RIN too low (2.30) to prepare a reliable sequencing library.

The low value of RIN is related to some degradation caused by the manipulation procedures for the isolation of the fresh intestinal epithelial cells (see the previous paragraph). However, as detailed in the Results, the percentage of mapping revealed that the RIN values were acceptable for our experiment.

### Preparation of the libraries and RNA sequencing data analysis

The 19 RNA-Sequencing directional libraries were prepared according to the NEBNext Ultra RNA library kit for Illumina sequencing, using poly-A mRNA magnetic isolation (New England Biolabs, MA, USA). The sequencing process was carried out in one single lane of an Illumina HiSeq 4000 platform, generating 150 base-paired end reads.

The quality of raw and cleaned sequences was checked using FastQC (http://www.bioinformatics.babraham.ac.uk/projects/fastqc/). Quality filtering and adapter removal were performed using a Trimmomatic v.0.35 [83]. The reads were aligned using STAR v.2.4.0.1 [84] to the chicken Ensembl reference genome (*Gallus gallus*v.*5*.0).

ReadCounter (http://www.genefriends.org/ReadCounter/references/) was used to quantify the number of read mappings on each gene locus using Galgal5 Ensembl (90) annotation coordinates.

### Differentially expressed genes and gene ontology analyses

Differentially expressed genes between CTRL and OMWW were evaluated by implementing a negative binomial distribution model in edgeR package (v.3.12.1) [85]. We filtered out features with a low number of reads per sample: one count-per-million in mover 50% of the samples was required to keep locus. Therefore, a total of 9162-filtered transcripts were used as input into edgeR. The analyses comprised 10 CTRL and 9 OMWW samples and we considered only the DEG with adjusted *p*-value (q-value) lower than 0.05 and absolute log Fold Change (logFC) > 1.0 as statistically significant.

Results were annotated using BioMart (http://www.ensembl.org/biomart/martview) and a curated list was used to carry out Gene Ontology (GO) enrichment and pathway analysis using ClueGO 3.2.0, a Cytoscape 3.3.0 plugin [86]. We considered GO-Term (Cellular Component – CC –, Biological Process – BP –, Molecular Function – MF –) and Kyoto Encyclopaedia of Genes and Genomes (KEGG) pathways with False Discovery Rate (FDR) < 0.05, using Benjamin-Hochberg correction as being statistically significant.

**Fig. 2:**
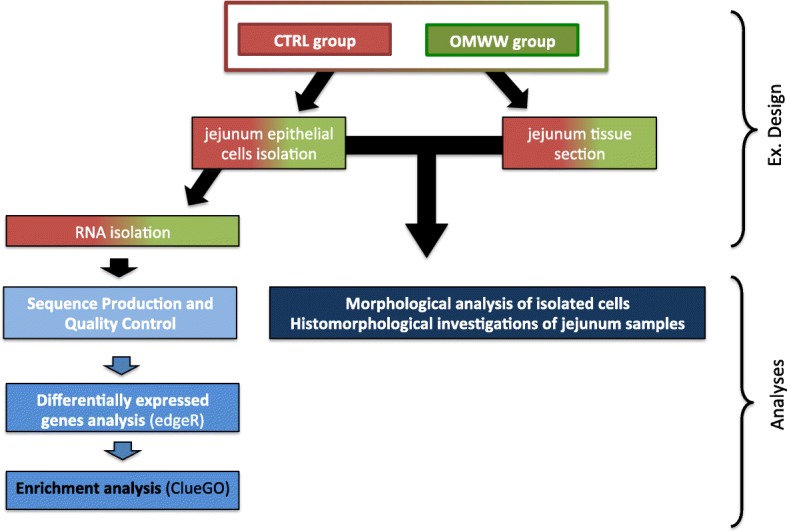
Experimental design and data analysis workflow

The workflow used in our study is shown in Fig. 2.

## Additional file

Additional file 1: **Table S1.** Significant differentially expressed genes (DEG), up regulated (logFC> 1) and down regulated (logFC<− 1) in OMWW group. Information contained in the table are significant Gene ID, GalgalEnsembl gene id (e.g. data ENSGALG00000041621); Transcript ID, GalgalEnsembl transcript id (e.g. data ENSGALT00000059872); Gene name, associated name of genes (e.g. LY6E); Gene description, description of gene name (e.g. Lymphocyte Antigen 6 Family Member); logFC, log Fold Change (e.g. 4,44E + 00); logCPM, log_2_ counts-per-million (e.g. 8,92E + 00); PValue, *p*-value evaluated in multiple testing (e.g. 3,96E-08); q-value, adjusted p-value (e.g. 2,41E-05). (XLSX 91 kb)

## Abbreviations

BP: Biological processes
CC: Cellular component
Cd: Crypt depth
CTRL: Control
DEG: Differentially expressed genes
FDR: False discovery rate
GO: Gene ontology
H&E: Haematoxylin & eosin
KEGG: Kyoto encyclopaedia of genes and genomes
logFC: Absolute log fold change
MF: Molecular function
OMWW: Olive mill wastewaters
q-value: Adjusted p-value
RIN: RNA Integrity number
SEM: Standard error of the mean
Vh: Villus height
